# Recurrent urinary tract infections and psychological burden: mechanisms and integrative perspectives

**DOI:** 10.3389/fmed.2025.1721343

**Published:** 2026-01-14

**Authors:** Tianyang Qian, Yining He, Ruxue Yan, Siyao Yu, Yuhan Chen, Weiming He

**Affiliations:** 1 Affiliated Hospital of Nanjing University of Chinese Medicine, Nanjing, China; 2 Nanjing University of Chinese Medicine, Nanjing, China

**Keywords:** endocrine system, immunity, infectious diseases, microbiome, neuroendocrine dysfunction, psychological burden, recurrent urinary tract infections (rUTIs), traditional Chinese medicine

## Abstract

Recurrent urinary tract infections (rUTIs) remain a global health concern with significant physical and psychological impacts, particularly in women. Recent evidence indicates a strong bidirectional association between rUTIs and psychological burden, yet the underlying mechanisms remain incompletely understood.This review integrates findings from biomedical and traditional Chinese medicine (TCM) perspectives to elucidate potential pathways linking rUTIs with anxiety and depression. Four major mechanisms—immune dysregulation, endocrine imbalance, microbiome alteration, and neuroendocrine dysfunction—are proposed to explain this complex interaction. In addition, TCM conceptualizes this relationship through the theory of the “coexistence of disease and depression syndromes” emphasizing that emotional regulation is a key determinant of both urinary and systemic health. By synthesizing these insights, this narrative review underscores the importance of integrative approaches in preventing and managing rUTIs while addressing concurrent psychological distress.

## Graphical Abstract

**Figure:**
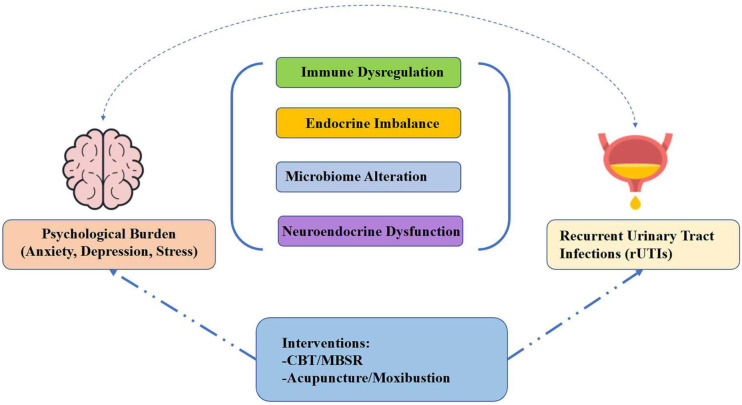
Graphical abstract illustrating the proposed bidirectional relationship between psychological burden and recurrent urinary tract infections (rUTIs). Psychological stress is suggested to influence rUTIs through four interrelated biological pathways: immune dysregulation, endocrine imbalance, microbiome alteration, and neuroendocrine dysfunction. Recurrence of UTIs may further reinforce psychological distress, forming a potentially self-perpetuating cycle. Integrative interventions—including cognitive–behavioral therapy (CBT), mindfulness-based stress reduction (MBSR), and selected traditional Chinese medicine (TCM) modalities—may help alleviate both psychological and urinary symptoms by targeting overlapping physiological and psychosocial factors. These relationships represent associations rather than confirmed causal pathways. Some icons used in this figure were obtained from Freepik (freepik.com) and Icons8 (icons8.com) and are used in accordance with the respective license terms.

## 1 Introduction

Urinary tract infections (UTIs) are among the most common infectious diseases worldwide. Approximately 60% of women experience at least one UTI during their lifetime, and 30%–40% develop recurrent urinary tract infections (rUTIs) (1). rUTIs are clinically defined as at least three UTIs within 12 months or at least two episodes within 6 months (2). In addition to the physical burden of recurrent dysuria, urgency, and frequency, rUTIs significantly impair quality of life and are often accompanied by psychological comorbidities such as anxiety and depression. Epidemiological studies indicate that anxiety disorders are among the most prevalent psychiatric conditions, with a global prevalence ranging from 2% to 29% (3). In China, 5.8% of women with UTIs were found to exhibit clinically relevant anxiety symptoms, whereas in rUTI patients, up to 68.8% experienced severe anxiety and 22.3% moderate anxiety (4, 5). These findings suggest a positive correlation between recurrence frequency and anxiety, indicating that anxiety levels increase as the rate of UTI recurrence increases.

The relationship between rUTIs and psychological burden is bidirectional. Symptoms such as recurrent urinary frequency, urgency, and dysuria often disturb daily life, predisposing patients to anxiety and depression, which in turn worsen the disease course and prognosis. Additionally, mental health issues may lead to behavioral changes such as impulsivity or impaired self-care abilities, thereby increasing the risk of rUTIs (6). Although the underlying mechanisms through which rUTIs and psychological factors mutually influence each other remain unclear, current evidence suggests four potential pathways: immune dysregulation, endocrine disturbance, microbiome dysbiosis, and neuroendocrine dysfunction. These pathways affect both neurotransmitter function and urinary tract physiology, creating a feedback loop that perpetuates both infection and psychological distress. In addition to biomedical explanations, traditional Chinese medicine (TCM) provides a complementary theoretical perspective. TCM attributes rUTIs to “lin syndrome,” in which emotional disturbances are important pathogenic factors. The theory of “coexistence of diseases and depression syndromes” emphasizes that unresolved emotional distress, such as worry, anger, or fear, can disrupt the flow of qi, damage organ function, and aggravate urinary symptoms. Previous reviews have largely focused on either biomedical mechanisms or TCM interventions independently. A combined perspective may better elucidate complementary pathways linking psychological burden with rUTIs, guiding integrative clinical strategies.

Therefore, this review aims to (1) summarize current evidence regarding the physiological mechanisms linking psychological burden with rUTIs, (2) discuss complementary insights from TCM theory and practice, and (3) propose potential integrative intervention strategies to guide future clinical research.

## 2 Materials and methods

**FIGURE 1:**
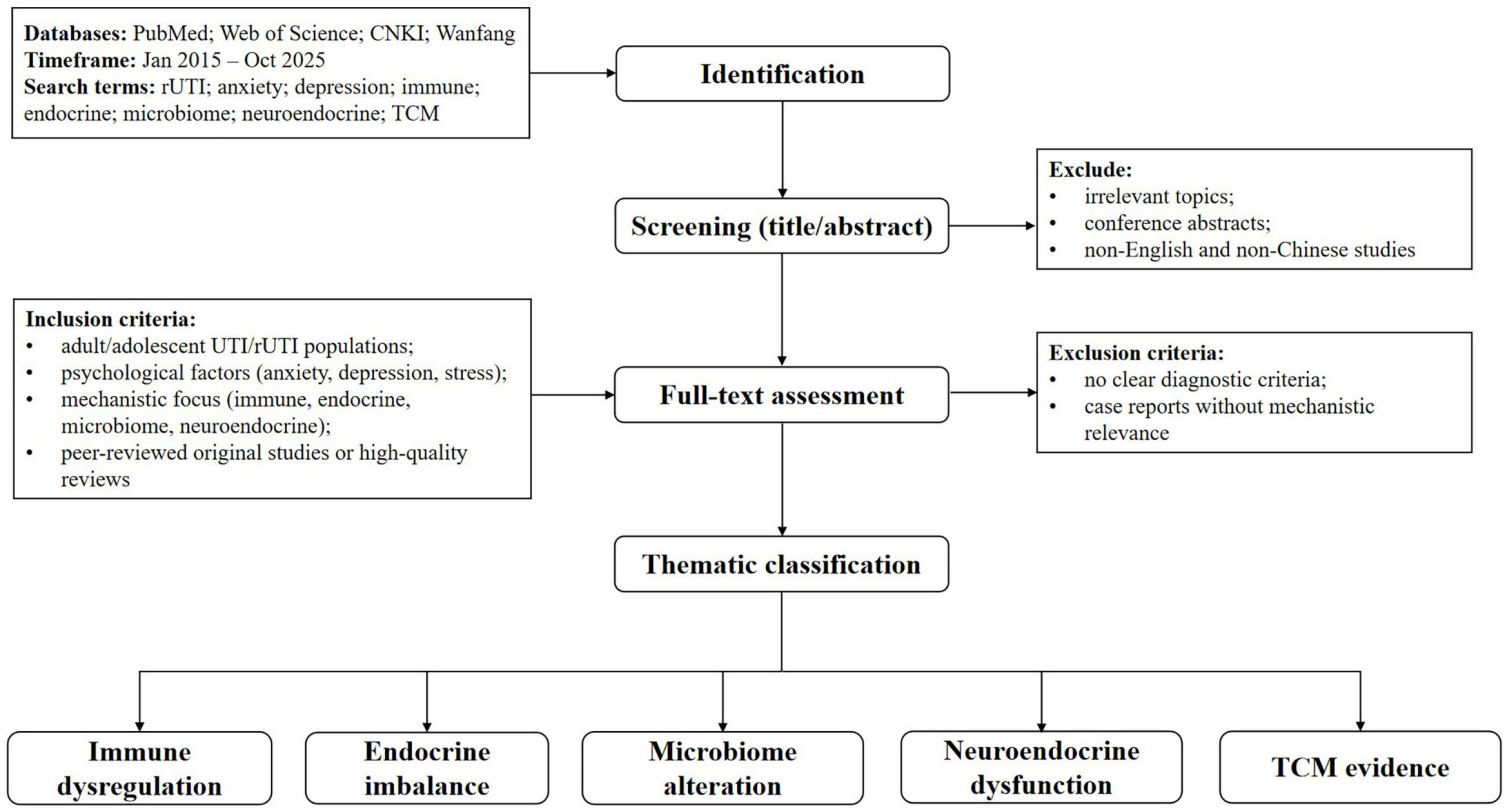
Study identification and selection process for this narrative review. A systematic search was performed across PubMed, Web of Science, CNKI and Wanfang Data for articles published between January 2015 and October 2025. Records were screened at the title and abstract level to remove irrelevant items (e.g., case reports, conference abstracts). Full texts were evaluated according to predefined inclusion and exclusion criteria (see section “2 Materials and methods”). Studies meeting thematic relevance for mechanisms linking recurrent urinary tract infections (rUTIs) and psychological burden (immune, endocrine, microbiome, neuroendocrine) and for traditional Chinese medicine (TCM) evidence were included in the final narrative synthesis.

To ensure comprehensive coverage of recent research, an electronic literature search was conducted in PubMed, Web of Science, CNKI, and Wanfang Data databases for studies published between January 2015 and October 2025.We searched each conceptual domain independently and reviewed articles containing any relevant combination of terms related to (1) rUTI, (2) psychological factors, (3) immune/endocrine/microbiome/neuroendocrine pathways, and (4) TCM or integrative frameworks. Domain-specific search terms included keywords such as “recurrent urinary tract infection,” “anxiety,” “depression,” “psychological stress,” “immune response,” “endocrine signaling,” “microbiome,” “neuroendocrine pathways,” “traditional Chinese medicine,” and “integrative medicine.” Approximately 3,692 records were identified across all databases. After removal of duplicates (*n* ≈ 1,250), roughly 2,442 titles and abstracts were screened. Approximately 700 full-text articles were reviewed in detail, and 120 studies met inclusion criteria and were incorporated into the qualitative synthesis (Figure 1). These numbers represent approximate counts in line with narrative review reporting standards.

Inclusion criteria comprised: (1) studies involving adult or adolescent populations with UTIs or rUTIs; (2) research examining psychological factors such as anxiety, depression or stress; (3) studies reporting immune, endocrine, microbiome or neuroendocrine mechanisms; and (4) peer-reviewed original studies or high-quality reviews published between 2015 and 2025.

Exclusion criteria included: (1) case reports without mechanistic relevance; (2) studies lacking clear diagnostic criteria for UTIs; (3) conference abstracts and opinion pieces; and (4) non-English and non-Chinese studies.

Two independent reviewers screened records and extracted data on study characteristics, psychological assessments, biological markers, and mechanistic outcomes. Discrepancies were resolved by discussion. Because this is a narrative review, findings were synthesized descriptively and organized into mechanistic domains.

## 3 Key findings

### 3.1 Immune dysregulation

The immune system serves as the primary defense mechanism against urinary tract pathogens, and its dysregulation plays a pivotal role in both infection susceptibility and psychological distress. Elevated urinary cytokine concentrations are closely associated with symptomatic UTIs (7). Numerous studies have reported increased levels of cytokines such as IL-1β, IL-6, TNFα, IL-8, and CXCL-10 during episodes of UTI (8). Compared with healthy individuals, active rUTI patients present significantly elevated concentrations of IL-1β, IL-8, IL-18, and MCP-1 in their urine, whereas the levels of anti-inflammatory cytokines such as IL-4 and IL-13 are markedly reduced (9), indicating a persistent proinflammatory state.

Concurrently, psychiatric conditions such as anxiety and depression are characterized by systemic immune activation. During rUTIs, the levels of proinflammatory factors such as IL-6, TNF-α, and C-reactive protein (CRP) are elevated. Once these molecules cross into the central nervous system, they can suppress the secretion of neurotransmitters, including serotonin and neuropeptide Y, disrupting neural balance and contributing to emotional and cognitive impairment, thereby reinforcing a vicious cycle (10). Zheng et al. (11) analyzed available data from participants in the UK Biobank and reported that patients with depression and anxiety had higher serum levels of CRP, IL-6, and other inflammatory markers. Additionally, the correlations between serum CRP levels and depression and anxiety were generally stronger in women than in men. This overlap suggests that inflammation serves as a shared biological substrate for both rUTIs and psychological disorders.

Emerging biomarkers further support this connection. Multiple studies have confirmed that anxiety patients have lower CD4+ and CD4+/CD8+ levels than healthy individuals do, indicating weaker immune function than healthy individuals do (12). PTX3 is a key component of the innate immune system and belongs to the pentraxin superfamily. Shelton et al. (13) reported that in fibroblasts from patients with severe depression and those with a depression subtype characterized by low PKA activity, PTX3 gene expression was 3.5 times higher than that in normal controls and non-depressed patients. Meanwhile, the expression of PTX3 in urinary epithelial cells serves as an early predictor of symptom severity and recurrence in rUTI patients. As the disease progresses, urinary PTX3 levels increase in rUTI patients compared with non-rUTI patients (14). Thus, immune dysregulation may represent the mechanistic bridge linking mental distress with recurrent infection cycles (Figure A Immune dysregulation).

### 3.2 Endocrine imbalance

Hormonal regulation is closely associated with both emotional stability and urinary tract health. Depression and chronic stress may reduce 5-HT receptor expression and influence the hypothalamic–pituitary–adrenal (HPA) and hypothalamic–pituitary–gonadal (HPG) axes, with potential downstream effects on estrogen levels (15). When 5-HT receptor expression is inhibited, estrogen levels correspondingly decrease.

#### 3.2.1 Estrogen and urogenital microbiota

Estrogen plays a vital role in rUTIs by regulating the urogenital microbiome. Estrogen receptors in women are primarily distributed in the urethral submucosal vascular plexus, urethral mucosa, and bladder trigone (16). Estrogen promotes the colonization of lactobacilli in the vaginal and urinary microbiota, thereby maintaining a protective microenvironment (17). Conversely, estrogen deficiency is thought to contribute to atrophy of the urethral and vaginal mucosa, capillary fragility, reduced secretion of protective substances and immunoglobulins, and depletion of vaginal lactobacilli. These changes allow pathogenic bacteria to colonize unchecked by lactate inhibition, thereby predisposing women to rUTIs (18).

Recent studies have demonstrated that estrogen supplementation restores lactobacillus colonization in postmenopausal women with rUTIs (19). Patients receiving vaginal estrogen experienced fewer urinary tract infections at 6 months (20).

#### 3.2.2 Estrogen and psychological regulation

In addition to its urogenital functions, estrogen has been linked to psychological regulation. Experimental data indicate that endogenous plasma estradiol levels are positively correlated with binding to cortical 5-HT(2A) receptors (21), suggesting a potential neuroendocrine contribution to mood modulation.

Additional neurobiological evidence further strengthens the link between estrogen signaling and psychological regulation. Experimental studies show that deficiency of estrogen receptor ERβ disrupts hippocampal BDNF signaling and increases 5-HT2A receptor activity, which closely involved in mood regulation (22). These findings provide a biologically plausible pathway linking estrogen deficiency with mood symptoms, although translation to human physiology remains preliminary.

Complementary mechanistic work further demonstrates that estradiol reduces 5-HT reuptake through ERβ-mediated downregulation of the plasma membrane monoamine transporter (PMAT), thereby increasing synaptic serotonin availability via the MAPK/ERK signaling pathway (23). This mechanistic evidence provides a biological explanation for the mood-modulating effects of estrogen and supports a potential interaction between estrogen signaling, serotonergic pathways, and stress-responsive neural circuits, but require confirmation in human studies (Figure B Endocrine imbalance).

### 3.3 Microbiome alteration

The gut-brain-bladder axis provides another essential pathway connecting psychological stress with urinary health. Anxiety can induce changes in the gut microbiota, which may, in turn, affect rUTIs. Studies have shown that, compared with healthy individuals, patients with anxiety disorders present higher levels of *Escherichia coli*, *Shigella*, *Clostridium*, and *Ruminococcus*, while the abundance of the dominant microbiota decreases (4). Changes in the gut microbiota can modulate susceptibility to rUTIs. Ghalavand et al. (24) confirmed that gut colonization by pathogenic strains can lead to endogenous urinary infection, based on molecular fingerprinting and genotyping of paired *Enterococcus faecalis* isolates from urine and fecal samples in symptomatic patients. Patients with rUTIs exhibit characteristics of gut microbiota dysbiosis, including reduced microbial diversity, decreased relative abundance of butyrate-producing microorganisms, and elevated plasma eosinophil chemokine-1 levels (25). Our team identified 56 metabolites associated with the *Ruminococcus* family UCG010 through Mendelian randomization. Analysis indicated that this bacterium downregulates N-acetyl-L-tyrosine levels, inhibits AhR-mediated inflammatory responses in macrophages and other cells, and subsequently promotes the development of urinary tract infections (26).

Alterations in the gut microbiota can also provide feedback and influence brain activity, as the gut microbiota can affect neurotransmitter levels, thereby influencing the central nervous system and anxiety-related behavior. The gut microbiota influences the nervous system through various mechanisms, including the production of neuroactive metabolites, modulation of the immune system, and activation of the vagus nerve (27). For example, gut microbiota dysbiosis reduces the expression of brain-derived neurotrophic factor (BDNF) in the cerebral cortex and hippocampus, leading to central nervous system dysfunction and the onset of anxiety (28). Microbiome disruption can modulate both immune responses and neurotransmitter metabolism, perpetuating the anxiety–infection feedback loop (Figure C Microbiome alteration).

### 3.4 Neuroendocrine dysfunction

Experimental and clinical observations suggest that anxiety may heighten neural excitability, which in turn can influence bladder smooth muscle responsiveness, although direct causal evidence in humans remains limited (29). Experimental evidence from the water avoidance stress (WAS) model shows that chronic psychological stress can damage the bladder mucosal barrier, causing urothelial shedding, reduced tight junction protein expression, and increased epithelial permeability (30), suggesting a potential mechanistic pathway that requires further validation in human studies.

#### 3.4.1 Central neuroinflammation

The paraventricular nucleus of the hypothalamus secretes corticotropin-releasing hormone (CRH) during stress responses. A human study examining stress and interstitial cystitis/bladder pain syndrome (IC/BPS) revealed altered CRHR expression in the bladder mucosa and submucosa, suggesting the CRH pathway may be involved in the mechanism of neurogenic inflammation in the IC/BPS bladder (31). Animal and translational studies further support the link between psychological stress and altered bladder sensory–motor function. Wood et al. demonstrated that chronic psychological stress induces marked lower urinary tract dysfunction, including shortened intervoid intervals, detrusor overactivity, and increased afferent nerve excitability. These stress-induced alterations were accompanied by enhanced sympathetic signaling and neuroinflammatory remodeling within the bladder wall, providing direct mechanistic evidence that psychological distress can heighten bladder sensitivity and amplify urinary symptoms (32). This experimental evidence aligns with clinical observations that anxiety frequently co-occurs with urgency and frequency, indicating that neuroendocrine dysregulation may be a key mediator linking psychological burden to rUTI susceptibility.

#### 3.4.2 Bladder sensory alterations under stress

Moreover, the bladder uroepithelium itself functions as a sensory organ. The bladder uroepithelium contains numerous receptors and ion channels and can release various neurotransmitters. A dense sensory nerve network is present in the subepithelial layer of the bladder, with some terminal fibers projecting into the urothelium and others terminating between muscle fibers. Neurotransmitters released from the urothelium are thought to influence the activity of afferent nerves in the bladder and play crucial roles in the transmission of bladder stimuli to the central nervous system. In animal studies, it has been confirmed that the bladder urothelium acts as sensory receptor cells. The bladder urothelium converts bacterial lipopolysaccharide (LPS) signals into neural signals through ATP-mediated pathways, leading to urinary frequency (33). Dysregulated neurotransmission under chronic stress may disrupt normal voiding reflexes and amplify bladder hypersensitivity, however, direct human evidence remains limited (Figure D neuroendocrine dysfunction).

**FIGURE 2:**
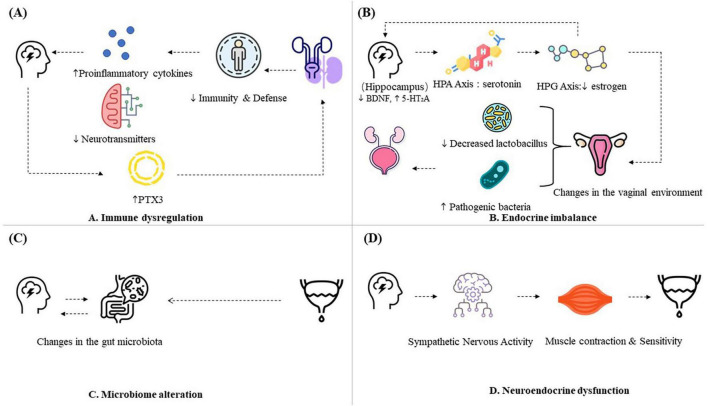
Mechanistic pathways of the bidirectional relationship between psychological burden and recurrent urinary tract infections (rUTIs). **(A)** Immune dysregulation: rUTIs are frequently characterized by heightened urinary cytokines and impaired innate responses. Psychological stress increases pro-inflammatory cytokines (IL-1β, IL-6, TNF-α) and decreases neurotransmitters, impairing host immunity and increasing susceptibility to rUTIs. Overlapping inflammatory signaling may contribute to vulnerability in both domains. **(B)** Endocrine imbalance: Chronic stress may activate the hypothalamic–pituitary–adrenal (HPA) axis and suppress the hypothalamic–pituitary–gonadal (HPG) axis, contributing to reduced estrogen levels. Lower estrogen is linked with vaginal atrophy, loss of Lactobacillus dominance, and increased susceptibility to uropathogen colonization. Experimental evidence suggests that estrogen also modulates hippocampal brain-derived neurotrophic factor (BDNF) signaling and serotonergic neurotransmission, offering a neuroendocrine basis for mood sensitivity. **(C)** Microbiome alteration: Psychological distress has been linked to gut microbiome dysbiosis, while rUTIs are associated with changes in urinary and gut microbial composition. These interactions may influence host immunity and urothelial physiology through the brain–gut–bladder axis. Most findings remain associative. **(D)** Neuroendocrine dysfunction: Preclinical and translational studies indicate that stress can alter bladder sensory–motor pathways, sympathetic activation, and mucosal signaling. Conversely, recurrent UTIs generate chronic pain and bladder irritation, which may intensify anxiety and depressive symptoms. Human mechanistic data remain preliminary. Some icons used in this figure were obtained from Freepik (freepik.com) and Icons8 (icons8.com) and are used in accordance with the respective license terms.

In summary, existing evidence indicates that immune, endocrine, microbiome, and neuroendocrine dysregulation are potential contributors within a multifactorial framework. Understanding these mechanisms provides a foundation for exploring complementary perspectives such as TCM, which emphasizes the role of emotional balance in disease prevention and recovery (Figure 2).

## 4 Traditional Chinese medicine perspective

Traditional Chinese medicine provides a framework that emphasizes the bidirectional influence between emotional disturbance and physical dysfunction, which aligns with several pathways proposed in modern psychoneuroimmune models of rUTIs. TCM has attached great importance to the regulation of emotions. It is believed that joy, anger, anxiety, thinking, sorrow, fear and fright are important causes of physical health problems. This is also known as the “internal injury caused by seven emotions,” and these emotions injure the five organs and cause disordered movement of qi. Zhu Danxi once said: If qi and blood are harmonized, all diseases will not arise; once you are depressed, you will be prone to many diseases. Therefore melancholy is the cause of most illnesses (34). Stagnation or depression of qi obstructs the normal transmission of fluids, resulting in bladder dysfunction. In modern society, individuals are often exposed to heavy social and psychological stress, which commonly leads to qi stagnation. In addition to depression caused by preexisting illness, illness itself can also provoke emotional depression, further impairing health. When the five qi are stagnant, all kinds of illnesses arise; this is depression caused by illness. rUTIs have a prolonged course, with a persistent and chronic nature that fails to resolve. Patients often endure repeated episodes of painful, urgent, or difficult urination, and in some cases, urinary incontinence, all of which severely impair quality of life. They are constantly anxious and fearful, with the disease and emotions mutually influencing each other, thereby triggering depression, such as anxiety and depression, and forming a vicious cycle of “disease-depression-disease” that repeatedly recurs. Although these terms do not directly correspond to measurable biomedical indices, their functional implications align with contemporary findings on stress-related neuroendocrine and inflammatory dysregulation.

To integrate TCM theory more directly with biomedical mechanisms, we mapped classical TCM syndromes onto corresponding physiological domains (Table 1). This mapping clarifies where conceptual alignment exists and where biomedical evidence remains preliminary. For instance, the TCM concept of liver-qi stagnation parallels chronic activation of the HPA axis and elevations in cortisol and proinflammatory cytokines observed in stress-related disorders. Although the physiological substrates differ, both frameworks emphasize impaired emotional regulation and sustained inflammatory signaling as contributors to urinary dysfunction. The TCM pattern of damp-heat in the bladder aligns with recurrent mucosal inflammation, reduced Lactobacillus stability, and increased Gram-negative colonization in rUTIs. This correspondence is supported by moderate clinical evidence and microbiome studies, although strain-level variability limits strong conclusions. Spleen deficiency with dampness maps onto impaired gut–brain axis integrity and weaker mucosal immunity; this is supported by emerging evidence demonstrating reduced short-chain fatty acid–producing bacteria and increased intestinal permeability in rUTI patients. However, findings remain preliminary. Kidney and liver yin deficiency corresponds to hypoestrogenic states and reduced vaginal microbiota stability, particularly in postmenopausal women. This mapping is supported by relatively consistent clinical evidence for estrogen deficiency as a risk factor for rUTIs, although TCM conceptualization focuses on systemic depletion rather than hormonal pathways.

**TABLE 1:** Mapping of traditional Chinese medicine (TCM) syndromes to biomedical correlates and supporting evidence.

TCM syndrome	Biomedical correlate	Supporting evidence
Liver-qi stagnation (emotional constraint, impaired flow of qi and fluids)	HPA-axis activation; chronic low-grade inflammation	Elevated cortisol and cytokines in stress-related disorders; neuroimmune links to urinary symptoms reported in stress-related bladder disorders
Damp-heat in the bladder	Microbiome dysbiosis; chronic mucosal inflammation	Reduced Lactobacillus, increased Gram-negative organisms; elevated IL-6, IL-8 in rUTIs
Spleen deficiency with dampness	Gut–brain axis disturbance; impaired immune tolerance	Altered short-chain fatty acid–producing bacteria; weakened mucosal immunity
Kidney and liver yin deficiency	Estrogen deficiency; vaginal atrophy; decreased microbiota stability	Lower estrogen in postmenopausal rUTIs; reduced Lactobacillus diversity
Disease–depression coexistence	Bidirectional psychosomatic feedback; neuroendocrine hypersensitivity	Associations between anxiety, pain sensitivity, and bladder sensory remodeling

Mapping of TCM syndromes to biomedical correlates and supporting evidence. These associations represent conceptual parallels rather than one-to-one physiological equivalence. Evidence levels range from clinical (immune and estrogen pathways) to preliminary (neuroendocrine–TCM intersections).

Recent scientific research has begun to provide empirical support for these traditional insights. Clinical studies show that Gualou Qumai Pill can enhance cellular immunity and reduce recurrence rates in rUTI patients (35), while Bazheng powder has been demonstrated to alleviate bladder inflammation in animal experiments (36). Experimental studies indicate that Dan Zhi Xiao Yao San can modulate M1/M2 microglial polarization in the hippocampus and ameliorate stress-induced anxiety-like behaviors (37). Acupuncture and moxibustion are widely used to relieve emotional disorders and regulate systemic balance. On the one hand, owing to its wide range of therapeutic effects, moxibustion, an external therapy in traditional Chinese medicine, has been used to treat emotional disorders such as anxiety and depression. Moreover, experiments have shown that moxibustion smoke has a good inhibitory effect on *Staphylococcus aureus, Streptococcus pyogenes, Escherichia coli, and Pseudomonas aeruginosa* (38). These findings bridge the psychological and physiological dimensions of rUTI management, showing that TCM interventions can impact both emotional regulation and urinary tract health.

Together, these mapped relationships show that TCM and biomedical perspectives are not parallel narratives but mutually informative frameworks. TCM emphasizes systemic pattern recognition and emotional–somatic coupling, while biomedical pathways specify molecular and cellular processes. When integrated, they highlight convergent mechanisms—such as stress-induced immune modulation and chronic inflammatory states—while acknowledging conceptual mismatch where direct physiological equivalence is not possible. This structured synthesis strengthens the interpretative model for rUTIs and supports the rationale for combining emotional regulation, immunomodulatory strategies, and microbiome-targeted interventions in clinical practice. By mapping TCM syndromes to biomedical correlates and identifying where evidence is strong, suggestive, or divergent, an integrated conceptual model can be formed that addresses both emotional and urinary tract dysfunction. This synthesis also highlights therapeutic implications: combining TCM herbal formulas, emotional regulation therapies, and standard antimicrobial or behavioral strategies may offer synergistic benefits for patients with recurrent infections.

## 5 Discussion

### 5.1 Key findings and mechanistic insights

This review highlights four primary biomedical pathways—immune dysregulation, endocrine imbalance, microbiome alteration, and neuroendocrine dysfunction—which may jointly mediate the bidirectional relationship between rUTIs and psychological burden. These mechanisms demonstrate the reciprocal relationship between mental health and infection recurrence. In parallel, TCM theories provide a complementary perspective, emphasizing the importance of emotional regulation and systemic balance in disease prevention and recovery.

Recent multidisciplinary studies increasingly support the intertwined nature of psychological distress and urinary tract health. Martin et al. (39) identified elevated levels of IL-6 and CRP are observed in patients with lower urinary tract symptoms accompanied by anxiety and depression. Meanwhile, Urakami et al. (40), Worby et al. (25) explored the existence of the gut-bladder axis, underscoring the significance of microbiome diversity reduction in UTI susceptibility. In addition, the psychosocial impact of recurrent urogenital infections described by Thomas-White et al. (41) reinforces the need to account for the psychological dimension in rUTI management. These studies align with our integrative framework linking psychological, microbial and urinary tract health. However, the evidence remains limited in several areas, including longitudinal research linking anxiety or depression directly to rUTI recurrence, and mechanistic trials on TCM interventions.

### 5.2 Evidence quality and methodological limitations

Although multiple biological pathways have been proposed to explain the relationship between psychological burden and rUTIs, the strength and consistency of the existing evidence vary substantially across domains. Immune and inflammatory pathways are supported by relatively robust human data, including large cohort studies and clinically measured cytokine profiles, which suggest associations between systemic inflammation, immune activation, and urinary tract symptoms. A recent comprehensive review on lower urinary tract inflammation and infection synthesized mechanistic and clinical findings showing that elevated urinary cytokines, epithelial barrier disruption, and impaired innate defense responses are consistently associated with rUTI susceptibility (42). However, the interpretation of these findings is constrained by several methodological issues. Most studies adopt cross-sectional or observational designs and rarely evaluate psychological stress, immune markers, and UTI outcomes concurrently, limiting the ability to test a psychoneuroimmune causal pathway. Heterogeneity in sampling timing (e.g., during acute infection, early recovery, or asymptomatic periods), variation in rUTI definitions, and demographic differences further complicate comparisons across studies. For instance, although increased IL-6 and IL-8 levels are often reported during active UTIs, a clinical study demonstrated that serum and urinary IL-6/IL-8 levels did not reliably distinguish upper from lower urinary tract infections in children (43). Evidence from biomarker research in pediatric UTI shows that urinary IL-9, IL-2, IL-8, and NGAL can distinguish children with true UTI from those with pyuria but no infection, whereas serum markers show lower accuracy (44). This discrepancy underscores how biomarker performance varies by sample type and may contribute to inconsistent immune profiles across rUTI studies.

Microbiome findings show even greater heterogeneity. Although several reports indicate reduced microbial diversity and depletion of butyrate-producing taxa in rUTI patients, strain-level results vary widely due to sequencing platform differences, antibiotic exposure, dietary variability, and inconsistent diagnostic criteria. Recent longitudinal metagenomic work found that women with rUTIs exhibit reduced gut microbial richness and significant depletion of butyrate-producing bacterial taxa compared with non-rUTI controls. However, the same study did not observe consistent differences in gut carriage of uropathogenic *Escherichia coli* between groups, suggesting that microbial composition alone may not fully explain recurrence (25). Instead, host–microbiome interaction and immune responsiveness may be more relevant. These inconsistencies indicate that microbiome-mediated mechanisms are promising but remain insufficiently validated. Another pilot study reported that female patients with recurrent cystitis have increased intestinal permeability and reduced gut microbial diversity compared to healthy controls, implying a compromised gut barrier and dysbiosis in recurrent infection cases (45). Nonetheless, small sample size, comorbid gastrointestinal conditions, and heterogeneous patient selection limit the generalizability of these findings. Collectively, while data from gut microbiome analyses provide a biologically plausible basis for a gut–bladder axis contributing to rUTIs, existing evidence remains preliminary and subject to methodological heterogeneity. Larger, longitudinal, and multi-omics studies are required to validate causality and identify specific microbial or host biomarkers predictive of infection recurrence.

Evidence for endocrine and neuroendocrine pathways relies heavily on animal models or small human studies. The interactions between estrogen deficiency, HPA/HPG axis dysregulation, serotonergic signaling, and bladder function are biologically plausible, but direct human causal data are sparse. Many effects—such as ERβ-mediated modulation of BDNF and PMAT—have been demonstrated primarily in rodents and require caution when extrapolated to clinical populations.

Overall, immune dysregulation is supported by relatively strong human evidence; microbiome alteration and endocrine pathways are supported by moderate and methodologically variable data; and neuroendocrine remodeling remains largely mechanistic or hypothesis-level. These differences should temper interpretation of the integrative model and highlight the need for longitudinal, multi-omics, and mechanistic clinical trials to strengthen causal inference.

### 5.3 Risk modifiers beyond psychological burden

Beyond psychological factors, several well-established contributors influence rUTI recurrence, including sexual activity, contraceptive methods, menopausal status, behavioral factors such as hydration and voiding habits, and antimicrobial resistance patterns. Although these variables were not the focus of this mechanistic review, they interact with psychological burden and should be considered when interpreting the current evidence base.

### 5.4 Clinical implications and integrative management

Given the multifaceted nature of the rUTI-psychological burden relationship, therapeutic strategies should concurrently target physiological and psychological domains. Psychological interventions such as cognitive-behavioral therapy (CBT) and mindfulness-based stress reduction (MBSR) can reduce anxiety and modulate immune responses, thereby potentially decreasing rUTI recurrence (46–51). From a TCM perspective, herbal formulas addressing damp-heat and emotional stagnation, acupuncture and moxibustion aimed at autonomic balance and qi flow, offer complementary benefits. Emerging experimental evidence, for example on formulas such as Gualou Qumai Pill and Bazheng powder indicates modulation of inflammatory cytokines and gut or bladder microbiota. Xu et al. (52) showed that Er Ding Er Xian combined with moxibustion significantly reduced recurrence rates and improved anxiety scores in clinical rUTI patients. However, high-quality clinical trials remain scarce. An integrative model combining antimicrobial measures, lifestyle and psychological support, and evidence-based TCM may provide the best outcomes in reducing recurrence, improving quality of life and addressing mental health comorbidities.

**FIGURE 3:**
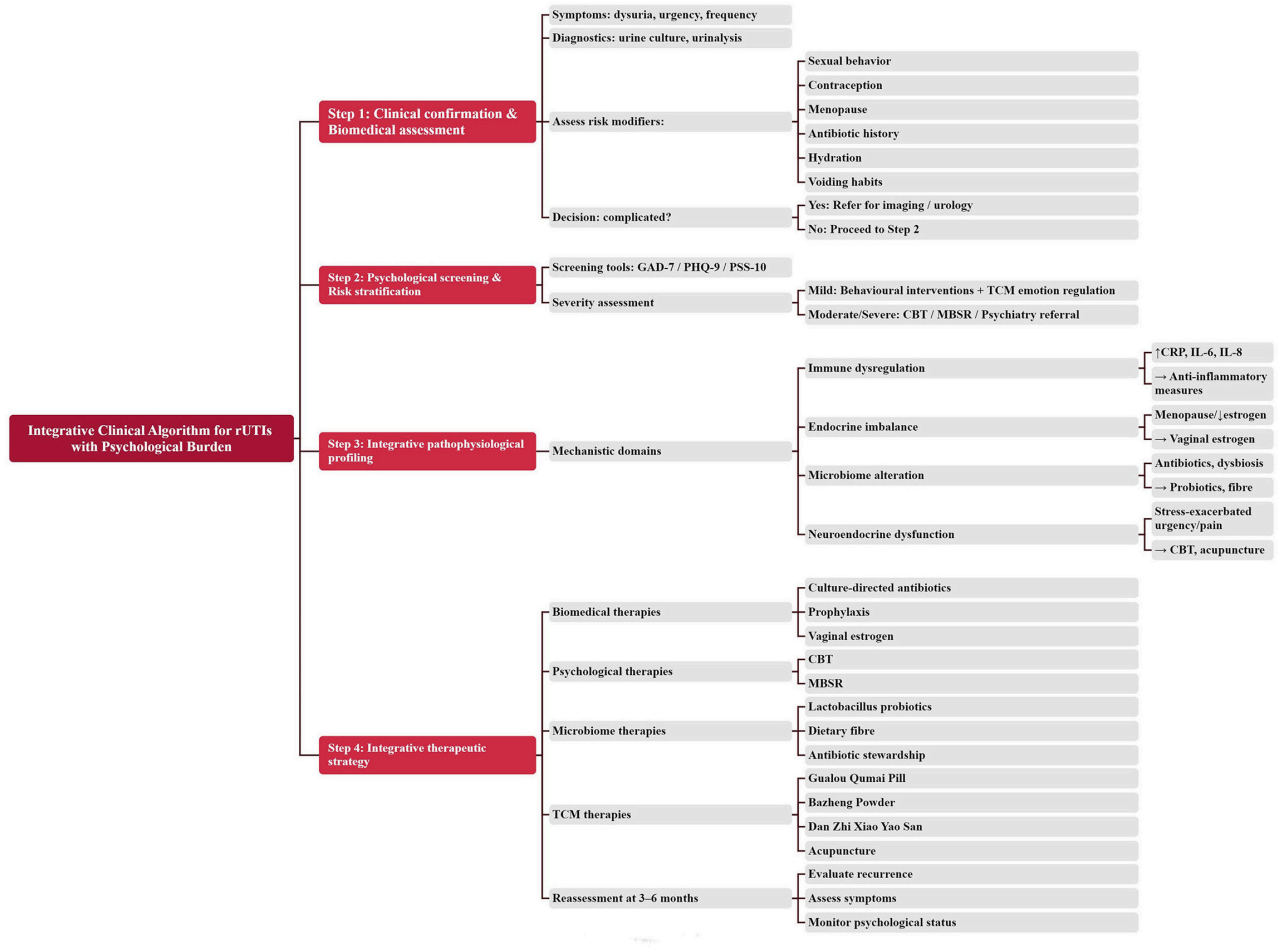
Integrative clinical algorithm for managing recurrent urinary tract infections (rUTIs) with concurrent psychological burden. Integrative clinical algorithm for evaluating and managing recurrent urinary tract infections (rUTIs) with concurrent psychological burden. The model outlines stepwise clinical decision-making incorporating biomedical assessment, psychological screening, mechanistic profiling, and combined therapeutic strategies. Step 1: Confirm infection; assess risk modifiers (sexual activity, menopausal status, antibiotic exposure, voiding habits). Step 2: Screen psychological burden using validated tools (Generalized Anxiety Disorder-7, Patient Health Questionnaire-9, Perceived Stress Scale-10). Step 3: Profile dominant mechanistic contributors (immune, endocrine, microbiome, neuroendocrine). Step 4: Implement integrative interventions including culture-directed antibiotics, hormonal or microbiome-directed therapies, psychological interventions (CBT, MBSR), and evidence-supported traditional Chinese medicine (TCM) modalities.

Given the multidimensional relationship between rUTIs and psychological burden, a structured integrative clinical algorithm is essential. Figure 3 presents a practical model combining biomedical assessment, psychological evaluation, risk stratification, microbiome-directed strategies, and evidence-supported TCM interventions.

#### 5.4.1 Step 1. Clinical confirmation and biomedical assessment

Objectives: confirm true infection, identify recurrence patterns, and rule out structural/urological abnormalities.

Key components:

- Symptom evaluation: dysuria, urgency, frequency, suprapubic pain.
- Diagnostic tests:
  ∘    Urine culture (>105 CFU/mL or symptomatic low-count bacteriuria),
  ∘    Urinalysis (leukocyte esterase, nitrites),
  ∘    Postmenopausal evaluation of urogenital atrophy.
- Risk modifiers assessment:
  ∘    Sexual behavior and post-coital exposure,
  ∘    spermicide/diaphragm use,
  ∘    menopausal status and hormone deficiency,
  ∘    Antibiotic history and resistance patterns,
  ∘    Voiding habits, hydration, constipation.

Decision node:

- If complicated UTI suspected (structural abnormality, stones, diabetes): refer for urologic imaging.
- If uncomplicated rUTI confirmed → proceed to Step 2.

#### 5.4.2 Step 2. Psychological screening and psychosocial risk stratification

Objectives: quantify anxiety, depression, and stress levels; determine whether psychological burden is contributing to recurrence.

Recommended tools (validated):

- GAD-7 for anxiety
- PHQ-9 for depression
- PSS-10 for perceived stress
- Optional: HADS, STAI

Decision node:

- Moderate–severe symptoms: refer to CBT, MBSR, or psychiatry as needed.
- Mild symptoms: integrate behavioral interventions + TCM emotional regulation strategies.

#### 5.4.3 Step 3. Integrative pathophysiological profiling

Objective: identify dominant mechanistic contributors (immune, endocrine, microbiome, neuroendocrine).

Suggested indicators:

- Immune dysregulation: elevated CRP, IL-6, IL-8; frequent inflammatory flares.
- Endocrine imbalance: menopausal status, low estrogen symptoms.
- Microbiome alteration: history of repeated antibiotics, constipation, dysbiosis symptoms.
- Neuroendocrine dysfunction: urgency worsened by stress; pelvic pain; hyperarousal.

Example interventions:

- Immune dysregulation: anti-inflammatory lifestyle strategies, TCM formulas for damp-heat.
- Endocrine imbalance: local vaginal estrogen; TCM formulas harmonizing liver–kidney.
- Microbiome alteration: probiotic lactobacilli, dietary fiber, TCM spleen-qi strengthening herbs.
- Neuroendocrine dysfunction: CBT; acupuncture; neuromodulation.

#### 5.4.4 Step 4. Integrative therapeutic strategy

Biomedical therapy:

- Targeted antibiotics based on culture,
- Prophylactic options when indicated,
- Vaginal estrogen for hypoestrogenic women.

Psychological interventions:

- CBT, ACT (acceptance-based therapy),
- Mindfulness-based stress reduction (MBSR).

Microbiome-directed therapy:

- Lactobacillus probiotics,
- Increased dietary fibre,
- Avoidance of unnecessary antibiotics.

TCM-based interventions (evidence-supported):

- Herbal formulas
  ∘    Gualou Qumai Pill (immune modulation, reduction of recurrence),
  ∘    Bazheng powder (reducing bladder inflammation),
  ∘    Dan Zhi Xiao Yao San (emotional regulation, microglial modulation).
- Acupuncture/moxibustion to improve autonomic balance and reduce anxiety.

Decision node:

- Reassess at 3–6 months → determine recurrence reduction, symptom improvement, and psychological stabilization.

### 5.5 Academic contribution and future directions

This review contributes academically by bridging conventional biomedical pathways with TCM theories, thereby offering a novel integrative mechanism model for rUTIs and psychological burden. Unlike previous reviews focused solely on microbial resistance or prophylaxis, our approach emphasizes the interplay of immune, endocrine, microbial and neuroendocrine systems within a psychosomatic and integrative medicine framework. This enriched perspective may stimulate future interdisciplinary research and inform holistic clinical strategies.

Future research should prioritize longitudinal cohort studies, mechanistic trials integrating psychoneuroimmunology and microbiome analysis, and high-quality RCTs assessing TCM plus psychological care in rUTI populations. Additionally, neuroimaging or metabolomic studies may clarify how emotional regulation therapies influence immune and urinary tract biology.

## 6 Conclusion

Recurrent urinary tract infections and psychological distress are interdependent conditions connected through immune, endocrine, microbiological, and neuroendocrine mechanisms. This review emphasizes the necessity of adopting a multidimensional treatment model that concurrently addresses mental health and urinary tract physiology.

From both biomedical and TCM perspectives, emotional balance and systemic harmony are essential for preventing disease recurrence. Future clinical practice should shift toward integrative models that combine pharmacological therapy, psychological interventions, and evidence-based TCM modalities. Such holistic strategies may ultimately reduce recurrence rates, improve quality of life, and enhance resilience against both infection and psychological stress.
